# Influence of Interferon-Alpha Combined with Chemo (Radio) Therapy on Immunological Parameters in Pancreatic Adenocarcinoma

**DOI:** 10.3390/ijms15034104

**Published:** 2014-03-07

**Authors:** Svetlana Karakhanova, Beate Mosl, Sabine Harig, Katharina von Ahn, Jasmin Fritz, Jan Schmidt, Dirk Jäger, Jens Werner, Alexandr V. Bazhin

**Affiliations:** 1 Department of General Surgery, University Hospital Heidelberg, 69120 Heidelberg, Germany; E-Mails: beate.mosl@med.uni-heidelberg.de (B.M.); sabine.harig@med.uni-heidelberg.de (S.H.); katharinavonahn@hotmail.com (K.A.); jasminfritz@web.de (J.F.); 2 National Centre for Tumor Disease, University Hospital Heidelberg, 69120 Heidelberg, Germany; E-Mail: Dirk.Jaeger@med.uni-heidelberg.de; 3 Department of General, Visceral, and Transplant Surgery, Ludwig-Maximilians-University, 81377 Munich, Germany; E-Mail: jens.werner@med.uni-heidelberg.de

**Keywords:** immunomonitoring. immunotherapy, interferon-α, CapRI, pancreatic carcinoma, Panc02 orthotopic model

## Abstract

Prognosis of patients with carcinoma of the exocrine pancreas is particularly poor. A combination of chemotherapy with immunotherapy could be an option for treatment of pancreatic cancer. The aim of this study was to perform an immunomonitoring of 17 patients with pancreatic cancer from the CapRI-2 study, and tumor-bearing mice treated with combination of chemo (radio) therapies with interferon-2α. Low doses of interferon-2α led to a decrease in total leukocyte and an increase in monocyte counts. Furthermore, we observed a positive effect of interferon-2α therapy on the dendritic cells and NK (natural killer) cell activation immediately after the first injection. In addition, we recorded an increased amount of interferon-γ and IL-10 in the serum following the interferon-2α therapy. These data clearly demonstrate that pancreatic carcinoma patients also show an immunomodulatory response to interferon-2α therapy. Analysis of immunosuppressive cells in the Panc02 orthotopic mouse model of pancreatic cancer revealed an accumulation of the myeloid-derived suppressor cells in spleens and tumors of the mice treated with interferon-2α and 5-fluorouracil. The direct effect of the drugs on myeloid-derived suppressor cells was also registered *in vitro*. These data expose the importance of immunosuppressive mechanisms induced by combined chemo-immunotherapy.

## 1. Introduction

Patients with pancreatic ductal adenocarcinoma are characterized by an especially poor prognosis reaching a five-year survival rate only in about 1% of cases and a median survival of four to six months. After resection, the five-year survival rate is at best 15% to 25% with additional adjuvant chemotherapy [1]. This cancer type represents globally one of the most aggressive malignancies. Adjuvant chemotherapy based on gemcitabine or 5-fluorouracil (5-FU) shows an increase in overall and disease-free survival [2–4]. However, the clinical impact of these chemotherapeutics remains modest. Therefore, new treatment options are urgently needed to improve the survival of patients with pancreatic cancer. Immunotherapy could be an attractive approach for treating such patients.

It has become increasingly clear that immunotherapy provides a promising option for cancer treatment [5], especially following the encouraging results from the Ipilimumab clinical study in malignant melanoma [6]. Furthermore, a combination of chemotherapy with immunotherapy can offer synergies and deliver improved efficiency in pancreatic cancer compared to current therapies [7]. As a result, combining standard chemotherapy with immunotherapy can provide a unique treatment of pancreatic cancer.

The first clinical phase III trial, dealing with the combination of 5-FU and interferon-2α (IFN), was the CapRI study [8]. IFN belongs to the group of type I interferons, which are already used in cancer therapy (e.g., malignant melanoma (metastatic [9] and adjuvant [10,11]), renal cell carcinoma (RCC) [12], and some other malignancies). IFN exhibits several features that might be of interest, especially for use in combination treatments of cancer, as there are: (i) direct inhibitory effects on tumor cell growth [13–17]; (ii) radio- and chemo-sensitizing effects [14,15,18]; (iii) anti-angiogenic properties [14,19]; (iv) enhancement of tumor immunogenicity (increase of MHC class I expression [20]); (v) modulation of the immune system: improving differentiation, maturation and function of dendritic cells (DC), enhancing the survival of T cells by expression of anti-apoptotic genes, generation of CD8^+^ memory cells, enhancing macrophage activities, and activating natural killer (NK) cells [20].

Clinical data from the CapRI study did not point at improved efficiency of the combination regime when compared to chemo-radiotherapy alone [21]. Despite this, the clinical outcome in both groups represent the best ever reported survival for patients with resected pancreatic cancer in randomized trials. In view of these findings, and the fact that the CapRI study revealed considerable toxicity of the combination treatment, a pilot trial (CapRI-2) was initiated to evaluate the influence of de-escalation of the CapRI regime on the immunological parameters by the omission of cisplatin and/or beam radiation [22].

The main aim of this research was the investigation of immunological effects of the type I interferon IFN and chemo-radio-immunotherapy with IFN and 5-FU in patients from the CapRI-2 trial, and in mice from the Panc02 orthotopic pancreatic carcinoma model.

## 2. Results

### 2.1. Human Study: Clinical Data

For the CapRI-2 study, 17 patients were recruited. All relevant clinical characteristics are summarized in Table 1. It should be pointed out that, during the study, the R1 resection had been redefined [23], thus, the majority of patients were adjusted from R0 to R1. The patients’ data, such as gender, resection type, tumor grade, TNM classification, and relapse did not show any significant differences between the several study arms (Table S1). Remarkably, six out of 17 patients were still alive and in good health with a maximal overall survival (OS) of 63 months (Table 1). The low number of patients in each study group unfortunately did not allow for a correct statistical analysis. Nonetheless, at least a certain number of two-year and five-year survivors could be calculated for each arm (Table S1). While no patient out of group A and C reached the five-year surviving edge, two patients out of group B (33%) successfully survived the five-year edge (Table S1).

### 2.2. Human Study: Immunological Effects of IFN Only

**Figure 1.**
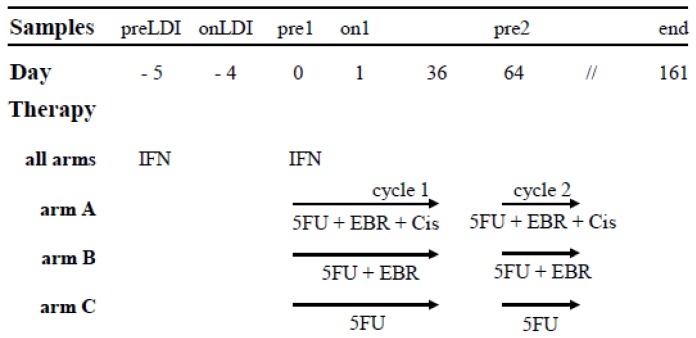
CapRI-2 therapy schema and time-points of blood withdrawal during the therapy. Blood samples were taken from the patients immediately before the first IFN injection (pre low-dose IFN (preLDI)), one day after (onLDI), immediately before the start of the first cycle of chemo-radio-immunotherapy or chemotherapy (pre1), one day after (on1), before the start of the second cycle of chemo-radio-immunotherapy or chemotherapy (pre2) and after a third cycle of the therapy (end). EBR—external beam radiation, Cis—cisplatin.

**Figure 2.**
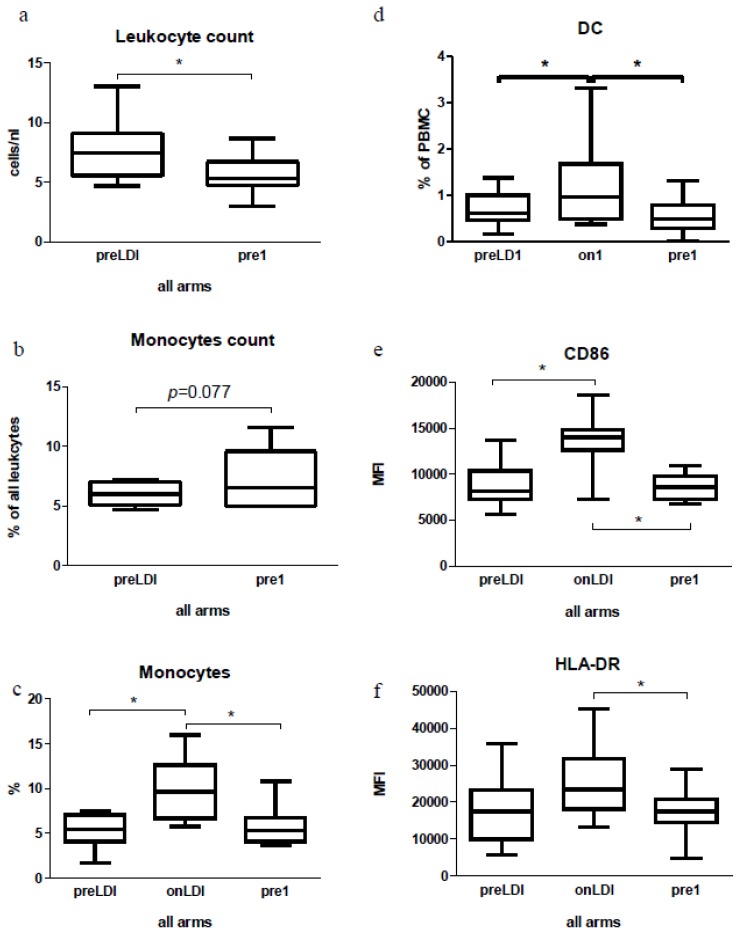
Analysis of leukocytes, monocytes and dendritic cells (DC) in peripheral blood of patients from the CapRI-2 study. (**a**) Absolute amount of leukocytes and (**b**) relative amount of monocytes during the course of IFN therapy (clinical chemistry), no data for onLDI; (**c**) the relative amount of monocytes in total leukocytes during the course of IFN therapy in all patients and in all therapeutic groups; (**d**) DC and their co-stimulatory markers during the course of IFN therapy (**e**) and (**f**). The immunological parameters of 17 patients were analyzed, * *p* < 0.05.

The initial phase of treatment was the same for groups A, B, and C (see Figure 1), which was why the impact of the first dose of IFN on immunological parameters could be evaluated for all 17 patients together. Treatment with the first low-dose of IFN led to a significant decrease in absolute amount of leukocytes (Figure 2a). From total leukocyte, the percentage of monocytes showed a high tendency to be increased (*p* = 0.077) (Figure 2b). Our FACS analysis also showed an increase in the cells within the monocyte gate (CD45^+^CD33^+^CD14^+^) immediately following a low-dose of IFN (Figures 2c and S1). Next, cells were additionally gated for dendritic cells (CD45^+^CD33^+^CD11c^+^CD14^−^) and investigated for the expression of HLA (human leukocyte antigen)-DR, CD80, and CD86 surface markers. We recorded an increase in DC following the first low-dose of IFN (Figures 2d and S1). For CD86 and HLA-DR, we also found a rise in their expression immediately after a low-dose of IFN (Figures 2e,f, and S2). A change in the CD80 expression was not obvious (data not shown).

We also examined whether preLD1 and pre1 points could differ in their lymphocyte count. We did not observe any differences between preLD1 and pre1 in this respect (data not shown). Afterwards CD8^+^, CD4^+^ and regulatory T cells (Treg, CD3^+^CD4^+^CD25^high^FoxP3^+^) in the lymphocyte gate were analyzed for their amounts and their phenotype and/or their activation status. We did not observe significant changes in relative numbers of T cell subsets (data not shown) in this instance. In addition, no differences were found in their activation status (based on the CD69 expression) after the first low-dose of IFN (data not shown). In a functional test, we did not observe an increase in granzyme B release during the course of IFN therapy after stimulation of patients’ PBMC (Peripheral blood mononuclear cells) with a CA19.9 peptide or with a MUC-1 peptide (data not shown).

In the current study, we also investigated an expression of the cytotoxic T-lymphocyte antigen 4 (CTLA4) on the surface of all CD4^+^ T cells following the first dose of IFN. We found no difference between the preLDI and onLDI in this instance: some patients responded to the low dose of IFN with an increase in CTLA4, some with a decrease and others showed no change (Figure S3). Additionally, we measured Treg in the CD4^+^ lymphocyte compartment; however, we did not observe any differences in their amount or in the CTLA4 or FoxP3 expression in these cells (data not shown).

**Figure 3.**
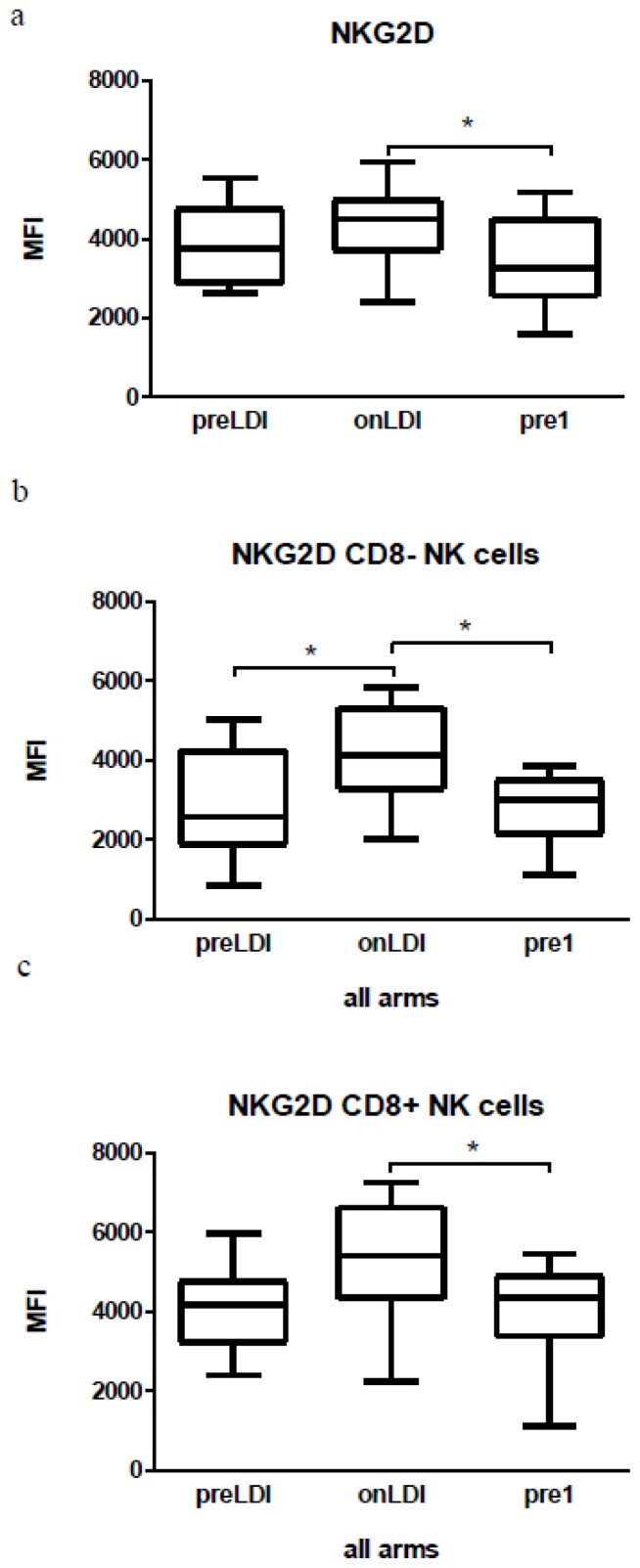
FACS analysis of NK cells and their activation state in the peripheral blood of patients. NK cells: expression of NKG2D on the surface of all CD45^+^CD56^+^ cells (**a**); CD45^+^CD56^+^CD8^−^ cells (**b**) and CD45^+^CD56^+^CD8^+^ cells (**c**) during the course of IFN therapy. The immunological parameters of 17 patients were analyzed, * *p* < 0.05.

Furthermore, we were interested in analyzing NK cells in the CapRI-2 patients. Whilst the total number of NK cells did not change, our FACS analysis revealed a significant increase in the activation status of NK cells, either in total NK cell population (CD45^+^CD56^+^) or in NKCD8^−^ or NKCD8^+^ cells (Figures 3 and S4). It should be noted that such activation dropped out to the time point pre1. An enhancement of activation indicated by upregulation of NKG2D receptors was detected immediately after a low-dose of IFN (Figures 3 and S4). Using chromium release assay against K562 cells, we investigated NK cell-mediated cytotoxicity. We could not demonstrate significant changes in cytotoxicity after the first low dose of IFN (data not shown).

### 2.3. Human Study: Effects of Chemo-Radio-Immunotherapy on Immunological Parameters

During the course of chemo-radio-immunotherapy, we observed a decreasing trend in the absolute amount of leukocytes (Figure S5a) and an increase in the monocyte subpopulation (Figure S5b) independent from the applied therapy. In addition, we detected a decrease in lymphocyte count during the chemotherapy/chemo-radiotherapy (ON1 *vs*. pre2) (Figure S5c). No difference was detected in the DC and in the expression of CD86, CD80, and HLA-DR on the surface of DC between the groups (data not shown). Furthermore, no difference was observed both in the relative numbers of T cell subsets or in the granzyme B release between the groups (data not shown). Following the second IFN injection, there was also no change detected in the total number of NK cells. However, we recorded an increasing trend in the NK cell activation independent from the applied therapy (Figure S6). There were no significant differences in the NK cytotoxicity, either during the course of IFN treatment or between the groups (data not shown).

### 2.4. Human Study: Effects of Chemo-Radio-Immunotherapy on Serum Cytokines

**Figure 4.**
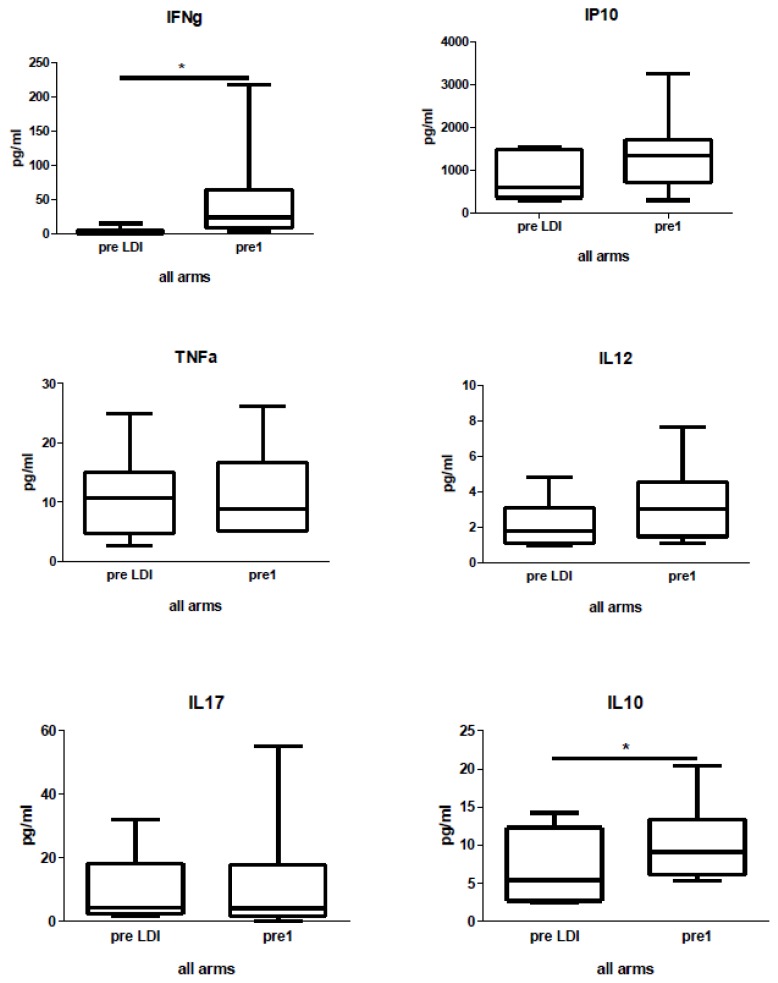
Cytokine level in the serum of patients before and after low-dose of IFN. The serum of 17 patients was analyzed, * *p* < 0.05.

Patients’ serum samples were taken at the following time-points: preLDI, pre1, on1, pre2, and end, for analysis of IFNγ, TNFα, IP10, IL12, IL17, and IL10 cytokines. We found an elevated level of IFNγ and IL-10 in the patients’ serum immediately following low-doses of IFN (Figure 4). High heterogeneity of patients in respect to initial serum level was observed for some cytokines (data not shown). We did not observe any difference in cytokine levels between the groups (data not shown).

### 2.5. Mouse Study: *In Vivo* Effects of Chemo-Immunotherapy on Immunological Parameters

**Figure 5.**
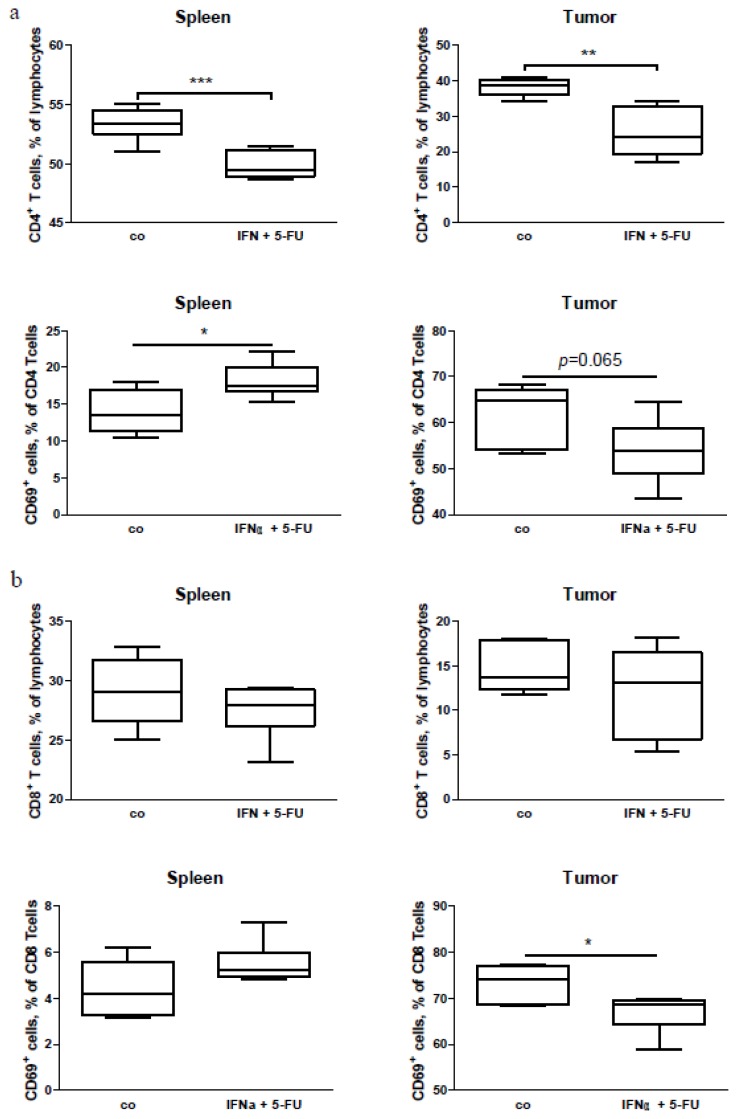
FACS analysis of lymphocytes in the spleen and tumors of Panc02-bearing mice treated with a combination of IFN and 5-FU (IFN + 5-FU), and of control-treated animals (co). (**a**) CD4^+^ T cells and their activation; (**b**) CD8^+^ T cells and their activation in the spleen and tumors of Panc02 tumor-bearing mice, respectively. Six to eight animals per group, * *p* < 0.05, ** *p* < 0.01 and *** *p* < 0.001.

**Figure 6.**
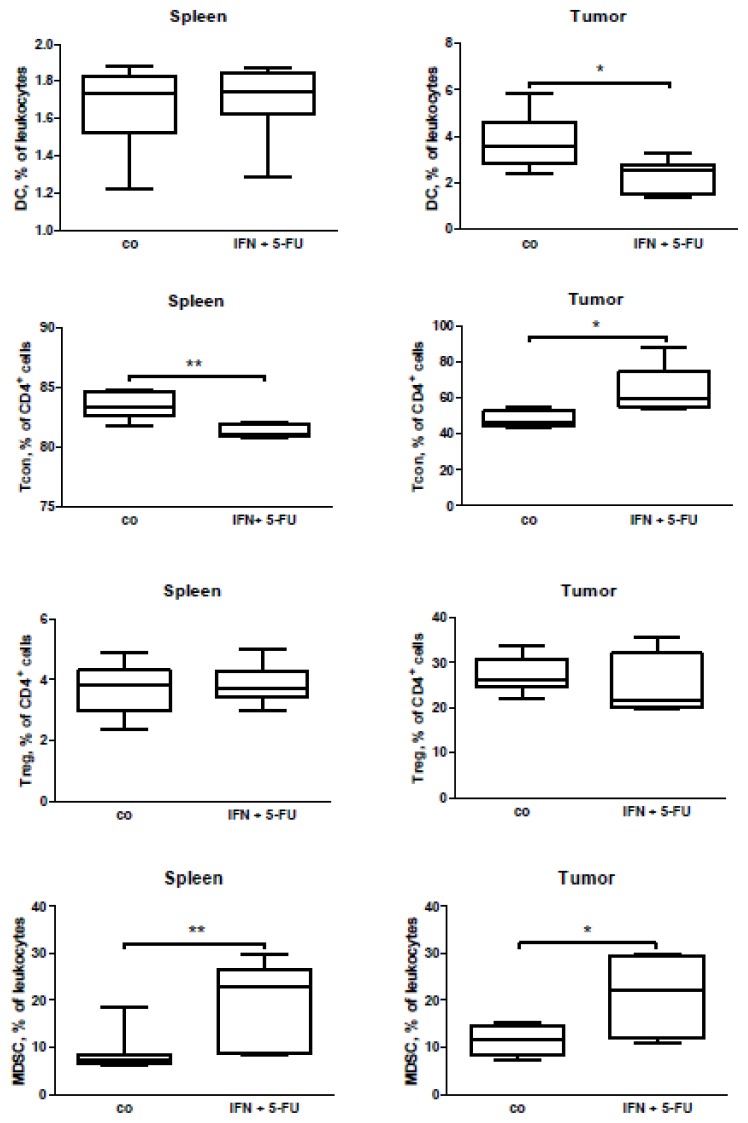
FACS analysis of DC, Tcon, Treg, and MDSC in the spleen and tumors of Panc02-bearing mice treated with a combination of IFN and 5-FU (IFN + 5-FU) and of contro-treated animals (co). Six to eight animals per group, * *p* < 0.05, and ** *p* < 0.01.

To extend our human *in vivo* data, we performed an immunomonitoring of tumor-bearing mice with pancreatic carcinoma treated with a combination of IFN and 5-FU. As the combination of IFN with chemo-radiotherapy is extremely toxic to mice (data not shown), we used only chemo-immunotherapy for the mouse treatment. As expected, the combination of IFN with 5-FU reduced the tumor growth significantly (Figure S7). The subsequent FACS (fluorescence-activated cell sorting, flow cytometry) analysis of splenocyte and tumor cell suspensions revealed a decrease in the CD4^+^ lymphocyte accumulation in the spleen and tumor (Figure 5a). With regard to the expression of the earlier activation marker CD69, we found an increase in the percentage of CD69^+^CD4^+^ T cells in the spleen following the combined treatment, and a tendency to a reduction of the cell percentage in the tumor (Figure 5a). For the CD8^+^ lymphocytes, we did not detect any change in percentage of the cells in either the spleens or the tumors, but a decrease in percentage of the activated (CD69^+^) lymphocytes (Figure 5b). Additionally, while there was no difference in the percentage of DC in the spleens of the tumor-bearing mice, a decrease in the percentage of DC was obvious in the mouse tumors following the treatment (Figure 6). It should be emphasized that the percentage of conventional T cells (Tcon, CD4^+^FoxP3^−^) had been reduced in the spleen and increased in the tumor of the tumor-bearing mice with pancreatic carcinoma (Figure 6), indicating a possible migration of Tcon to the tumor site.

Investigation of the immunosuppressive arm during the combined therapy revealed that IFN in combination with 5-FU increased an accumulation of myeloid-derived suppressor cells (MDSC) in both the spleens and in the tumors of the treated tumor-bearing mice. There was, however, no effect on the Treg (CD4^+^CD25^+^FoxP3^+^) percentage (Figure 6). It should be stressed that in the control (untreated) group of mice, we found a promising negative correlation of tumor growth with CD4^+^ lymphocyte accumulation and an upward trend for a positive correlation of tumor growth with MDCS accumulation (Table 2). This demonstrates that MDSC were able to diminish the accumulation of CD4^+^ cell in the tumors. However, in the IFN + 5-FU treated group of mice, we could not find such a correlation (Table 2).

### 2.6. Mouse Study: *In Vitro* Effects of Chemo-Immunotherapy on Immunological Parameters

**Figure 7.**
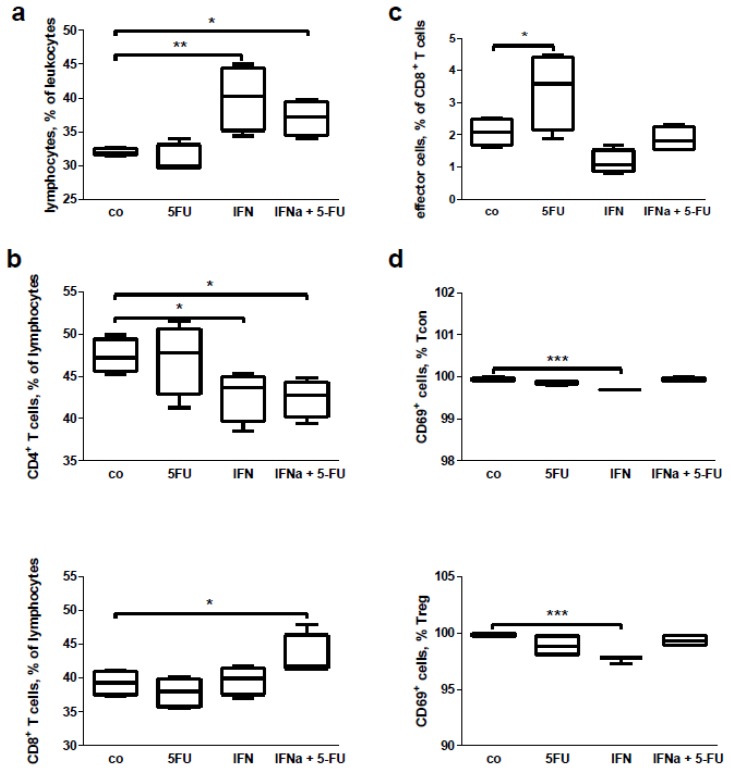
*In vitro* effects of therapies with IFN or 5-FU treatment only or their combination on splenocytes from tumor-bearing mice: FACS analysis of (**a**) lymphocytes; (**b**) CD4^+^ and CD8^+^ lymphocytes; (**c**) CD8^+^ effector cells; (**d**) CD69 expression on the surface of Tcon and Treg. Results of four independent experiments are presented, * *p* < 0.05, ** *p* < 0.01 and *** *p* < 0.001, co—control treated mice.

**Figure 8.**
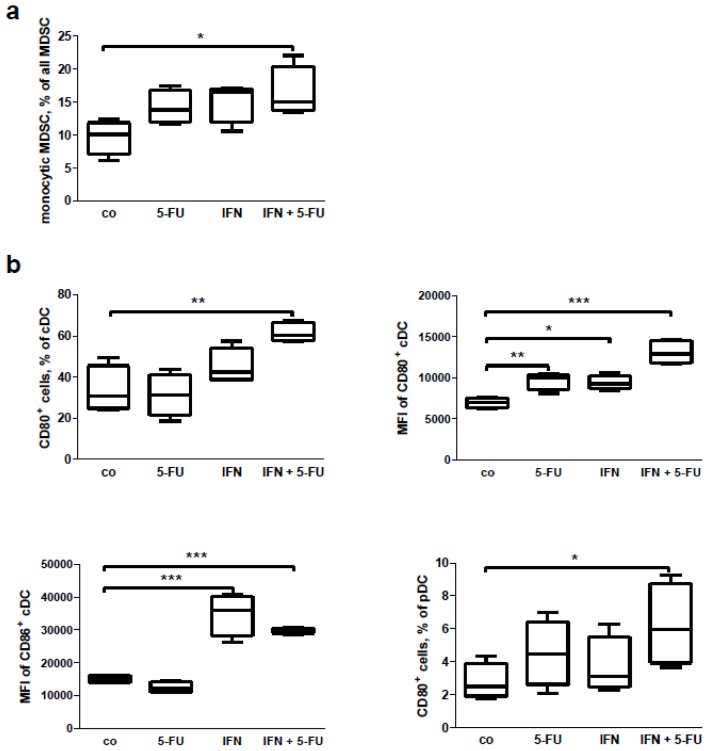
*In vitro* effects of therapies with IFN or 5-FU only or their combination on splenocytes from tumor-bearing mice: FACS analysis of (**a**) monocytic MDSC and (**b**) cDC and pDC. Results of four independent experiments are present, * *p* < 0.05, ** *p* < 0.01 and *** *p* < 0.001, co—control treated mice.

Finally, we wanted to examine whether the IFN or 5-FU, or combined IFN + 5-FU therapy had a direct effect on immune cells. To do this, we isolated splenocytes from the tumor-bearing mice and cultivated these *in vitro* for 24 h with the drugs or with the compound combination. FACS analysis of the cultivated splenocytes revealed an increase in the percentage of lymphocytes in the leukocyte gate after the IFN treatment alone and in combination with 5-FU, but not after 5-FU treatment only (Figure 7a). It is interesting to note that the combined treatment changed the CD4^+^/CD8^+^ ratio, decreasing the CD4^+^ cell percentage and increasing the CD8^+^ one (Figure 7b). Treatment of the cells with IFN alone led to an increase in the percentage of CD8^+^ effector cells (Figure 7c). No influence on lymphocyte subpopulations (*i.e*., naïve, effector, effector-memory or central memory cells) was detected (data not shown). In addition, no *in vitro* effects of the treatments were found on the percentage of Treg, conventional T cells and macrophages (data not shown). However, IFN-treatment decreased the earlier activation of Tcon and Treg based on expression of the CD69 surface marker (Figure 7d). 5-FU treatment alone did not change the immunological parameters assessed. While MDSC as a whole and granulocytic subpopulation of these cells did not show changes after the treatment (data not shown), the percentage of monocytic MDSC was increased following IFN + 5-FU treatment (Figure 8a). In respect of DC, we found an upregulation of CD80 and CD86 co-stimulatory molecules on the surface of cDC and an increase in expression of CD80 on pDC following different treatments (Figure 8b); the percentage of DCs was not affected (data not shown).

## 3. Discussion

The results of the CapRI-1 trial did not feature a survival benefit for patients with pancreatic cancer by adding interferon Type I (IFN) to the chemotherapy regimen. The small group of recruited patients in the CapRI-2 trial unfortunately does not permit a precise and statistically relevant investigation of survival data. However, the particularity, that five-year survivors were only found among patients of group B, should arouse scientific interest and encourage new study designs. The benefit of patients in this group could be traced back to either the omission of cisplatin, or the higher amount of patients with R0 resection.

Both in patients and in mice we observed a decrease of total leukocyte count. This result was in accordance with the IFN effects found in both healthy subjects [24] or in patients treated with IFN [25,26]. This could indicate that the decrease in leukocyte count is a response to IFN. Despite total decrease in leukocyte count, we observed an increase in the monocyte count between preLD1 and pre1. This was additionally supported by the data from FACS analyses, which demonstrated a significant increase in monocytes already being between preLD1 and onLD1. In our case, this upregulation was significant amongst all 17 patients following the first IFN doses (without an additional therapy); the same tendency after second IFN injection was observed between all three groups. Therefore, we speculate that IFN was primarily responsible for this effect without an additional impute of cisplatin, radiation or even 5-FU. A similar effect of IFN on monocyte count has also been described for healthy individuals [24]. Even in our data gained from the *in vitro* mice study, the major immunological effects were due to the presence of IFN. Thus, our data and those from others reflect the general effects of IFN treatment on leukocytes.

In the current work, we documented the positive effect of IFN therapy on the DC subpopulation and their activation both in human and mice systems. The impaired activity of DC, indicated by reduced allostimulatory activity by *ex-vivo*-generated DC, seems to be common to pancreatic cancer patients [27]. Therefore, an increase in the DC activation could be favorable for the patients’ anti-tumor immunity. In the present work, this effect was significant among all 17 patients after the first IFN dose and as we did not observe further significant differences during the course of therapy or between the therapeutic groups, we conclude that IFN itself (without subsequent chemotherapy) was responsible for this activation. At present, there are no reliable *in vivo* data from other IFN therapeutic trials that demonstrate an IFN impact on DC activation. However, our results were supported by our *in vitro* observations showing the IFN + 5-FU influence on the DC activation status, reflected by upregulation of co-stimulatory molecules. These results were supported by observations *in vitro*, where IFN increased alongside the expression of the DC co-stimulatory molecules [28–30].

T lymphocytes were extensively investigated in this research. We did not observe any effect of IFN on CD4^+^ and CD8^+^ T cells (including their subpopulations) *in vivo* both in the human and in the murine system. However, direct IFN treatment alone increased the percentage of effector CD8^+^ cells and in combination with 5-FU improved the CD8^+^/CD4^+^ ratio. Therefore, we cannot exclude that the same positive effects of IFN *in vivo* might be observed in other experimental settings.

Following the encouraging data from the Ipilimumab clinical study in malignant melanoma [6], the suppression of CTLA4 expression on lymphocytes could be a new strategy in the therapy of some immunogenic cancers. In this study, we did not find an influence of IFN or chemo-radio-immunotherapy on CTLA4 expression. Therefore, the immune therapy of pancreatic cancer with monoclonal antibody against CTLA4 (*i.e*., Ipilimumab) could be a new option for ameliorating a combination therapy.

The augmentation of NK cell activity is important for antitumor response. This has been documented in several IFN trials, as well as in other types of immune stimulatory therapies (for review see [31]). In our work, we proved an increase of the activated NK cell amount following the first IFN injection. This was in line with data from other groups, where increased NK activation was associated with stimulatory immunotherapy [32,33]. Additionally, earlier observations showed that the increased NK cells mediated cytotoxic activity following IFN treatment [34].

In this study, we demonstrated an increased serum level of IFN-γ and IL-10 after IFN therapy. IFN-γ acts as a proinflammatory cytokine important to the action of T cells and promotes anti-tumor immunity [35]. In opposite to IFN-γ, IL-10 possess anti-inflammatory properties that have an effect on keeping a “IFN-γ/IL-10 balance” and can be the indicator of the action of Treg [36]; however, it can also potently activate B lymphocytes [37]. Therefore, based on the cytokine serum level, we concluded that the unspecific IFN immunotherapy could potentially modulate both pro- and anti-inflammatory mediators in patients with pancreatic carcinoma.

In this clinical trial, the immunosuppressive arm was not accurately assessed. Only Treg were traced in this study, but there were no effects on these immunosuppressive cells. These data on Treg were confirmed by our *in vitro* data showing the absence of IFN + 5-FU effects on Treg. Effects of IFN on Treg in clinical studies have been investigated in cases of renal RCC [38] and melanoma [39]. While in RCC patients, the percentage of Treg decreased immediately after IFN treatment, the amount of cells recovered later as treatment processed [38]. In another clinical study with melanoma patients, the combination of IFN with tremelimumab (an antibody against CTLA4) induced an increase in Treg percentage. The authors assumed that this effect was mainly due to the CTLA4 blockage [39]. Therefore, effects of IFN on Treg should be investigated further.

In regard of MDSC, the orthotopic mouse model of pancreatic cancer was characterized by higher accumulation of MDSC in the tumors [40]. Furthermore, the MDSC accumulation was elevated after chemo-immunotherapy in both mouse spleens and tumors; this accumulation had a positive correlation with tumor growth and simultaneously a negative correlation with the amount of CD4^+^ cells. Our assumption is that this treatment interacts on the MDSC/CD4^+^ T cell interplay and in turn on tumor growth. *In vitro* effects of combined drug administration was shown only for monocytic MDSC. These data point to the importance of immunosuppression (through MDSC) induced by combined chemo-immunotherapy.

Additionally, in this work, we found a high heterogeneity in the initial level of some immunological parameters (CTLA4 expression, serum cytokine level, NK-mediated cytotoxicity and antigen specific cytotoxicity) prior to IFN therapy in the individual patients. This could potentially have led to the different responses to IFN therapy. We suggest initial immunological stratification of patients for immunotherapeutical trials. This would allow for precise immunomonitoring and improving the response to the therapy.

Summarizing our human and mouse *in vivo* and *in vitro* studies allowed us to conclude that the unspecific immunotherapy with interferon type I of patients with pancreatic carcinoma induced not only the activation of anti-tumor immunity, but, also, immunosuppressive mechanisms. This might be a reason for the absence of clinical benefit for patients treated with the combined therapy in the CapRI-1 study [41,42]. This hypothesis needs more experimental efforts, which should be conducted in future immunotherapeutic trials and combined with precise immunomonitoring.

## 4. Experimental Section

### 4.1. Patients Characteristics and Study Set up

Patients over the age of 18 years old with biopsy-proven completely resected (R0 or R1 [23]) pancreatic adenocarcinoma were eligible for participation in the CapRI-2 study [22]. The final protocol was approved by the ethics committee of the University of Heidelberg, Medical School, Heidelberg, Germany (AFmu-071/2008). Trial Registration: Current Controlled Trials ISRCTN79802092. Before commencing the therapy, patients were informed of the nature and risks of the study by a physician and provided their written consent. According to the CapRI-2 protocol [22], patients in study group A received a combination of 5-FU, cisplatin and external beam radiation (EBR), including administration of IFN (CapRI regimen). The first de-escalation step was group B, where cisplatin had been omitted (CapRI-light). The second de-escalation step was group C, where both cisplatin and EBR were left out (CapRI ultra-light) (Figure 1). For final data evaluation of the immunological parameters, 17 patients were presented. Patients’ characteristics are summarized in Table 1.

### 4.2. Time Point of Analysis and Sample Preparation

Blood samples were taken from the patients immediately prior to the first IFN injection pre low-dose IFN (preLDI), one day after (onLDI), immediately before the start of the first cycle of chemo-radio-immunotherapy or chemotherapy (pre1), one day after (on1), before the start of the second cycle of chemo-radio-immunotherapy or chemotherapy (pre2), and after the third cycle of the therapy (end) (Figure 1). Peripheral blood mononuclear cells (PBMC) were isolated from heparinized blood samples by Ficoll density gradient centrifugation. The cells were frozen in 10% DMSO (Sigma, Deisenhofen, Germany) and 20% fetal calf serum (PAA Laboratories, Cölbe, Germany), and the serum samples were stored at −80 °C. EDTA blood was used immediately for flow cytometric analysis. For cytotoxicity assays, the monocytes were removed after thawing by adherence to plastic. Leukocyte count (all time points except onLDI) was done in the routine analysis center of the Department of Clinical Chemistry University Hospital Heidelberg according to the standard operating procedure.

### 4.3. Cell Culture

The murine pancreatic adenocarcinoma cell line Panc02 was originally from Corbett *et al*. [43]. Cells were cultivated in a RPMI-1640 medium with 10% fetal calf serum, 100 U/mL penicillin and 100 μg/mL streptomycin, (PAA Laboratories, Cölbe, Germany). Cells were cultivated at 37 °C and 5% CO_2_ and routinely checked for mycoplasma contamination.

### 4.4. Mice

C57BL/6 mice (six to eight weeks) were purchased from Charles River (Sulzfeld, Germany) and kept under specific pathogen-free conditions in the animal facility of Heidelberg University (IBF, Heidelberg, Germany). Animal experiments were carried out following the approval of the German authorities (35-91585.81/G-184/11).

### 4.5. Orthotopic Mouse Model of Pancreatic Carcinoma

Syngeneic pancreatic carcinoma cells were injected orthotopically in C57Bl/6 mice, as described elsewhere [40]. Mice were narcotized with isofluran/O_2_ inhalation. After achieving surgical tolerance (stage III_2_), mice were opened with an abdominal section. A volume of 5 μL of mycoplasma-free Panc02 cells in a concentration of 2 × 10^5^ cells/mL PBS were injected into the pancreatic head with a 25 μL gastight syringe (Hamilton, Reno, NV, USA). The injection site was clamped for about 30 s after removal of the syringe. The tissue was carefully returned to its original position and all layers of the wound (peritoneum, muscles, and skin) were sutured with synthetic absorbable suture material (polysorb 6-0, (Tyco Healthcare, Neustadt, Germany)). Mice were treated i.p. with a combination of murine recombinant IFN (type I interferon) (1 × 10^4^ units per mouse/day) and 5-FU (35 mg/kg/day) on days five, seven, and nine. Tumors and spleens were harvested four weeks after Panc02 cell implantations and used for the flow cell biology analysis.

### 4.6. Flow Cell Biology Analysis of Human Blood Samples

Human leukocytes were characterized using following fluorescence labeled anti-human antibodies, as described elsewhere [44]: CD45 (for all leukocytes), CD3, CTLA4, FoxP3, CD25, CD56, NKG2D, CD69, CD8, CD19, CD45RA, CCR7, CD27, CD33, CD14, CD11c, CD80, CD86, and HLA-DR (all BD Biosciences, Heidelberg, Germany). For FACS analyses, antibodies were placed into a 5 mL polystyrene round bottom tube and 100 μL blood were added to the tube, which was briefly shaken and incubated for 15 min at room temperature. Afterwards, 2 mL 1× BD FACS Lysing Solution (BD Biosciences, Germany) was added and further incubated for another 10 min at room temperature, with centrifugation following at 200× *g* for 5 min. The supernatant was removed, the pellet re-suspended in 2 mL Cellwash solution (BD Biosciences, Heidelberg, Germany) and centrifuged again. Cells were re-suspended in 250 μL Cellwash and measured on a BD FACS Canto II flow cytometer (BD Bioscience, Heidelberg, Germany). FoxP3 cytoplasmatic staining was performed with a FoxP3-Buffer Set and FoxP3-PE antibodies (both from BD Bioscience, Heidelberg, Germany), according to the manufacturer’s instructions. Briefly, 400 μL of EDTA-blood was placed into the tube and 1 mL Versalyse (Beckman Coulter, Krefeld, Germany) was added; the mix was shaken and incubated for 10 min, then centrifuged for 5 min with 200× *g* and re-suspended in 2 mL of Stain buffer (BD Biosciences, Heidelberg, Germany). After centrifugation, the supernatant was removed and the pellet re-suspended in 100 μL Stain Buffer. Antibodies for cell surface proteins were added for 20 min (room temperature); cells were washed twice and fixed with a fixation buffer. For permeabilization, cells were supplemented with 0.5 mL of buffer C and incubated for 30 min. After being rinsed twice, 10 μL of FoxP3-PE antibodies were added and the cells were incubated for 30 min. After being rinsed twice again, cells were re-suspended in 250 μL Stain Buffer and measured on a BD FACS Canto II flow cytometer (BD Bioscience, Heidelberg, Germany). Data were analyzed with FlowJo software (Tree Star, Inc., Ashland, OR, USA).

### 4.7. Flow Cell Biology Analysis of Murine Splenocyte and Tumor Samples

FACS analysis of murine leukocytes was performed as described elsewhere [40] and will be described later in detail. Single cell suspensions of tumors and spleens were prepared in the Stain Buffer (PBS supplemented with 1% mouse serum and 1 mM EDTA). The cells were then counted and the density adjusted to 4 × 10^7^/mL. The unspecific binding caused by the Fc receptors was blocked by incubating the cells with anti-mouse CD16/CD32 antibody (1 μL for 2 × 10^6^ cells) at 4 °C in the dark for max 20 min. Then, a 50 μL cell suspension (2 × 10^6^ cells) was incubated with 50 μL of the Stain Buffer containing various monoclonal antibodies at 4 °C, also in dark conditions for max 20 min. A Foxp3 Staining Buffer set was used for intracellular staining according to the manufacturer’s instructions. Acquisition was performed by flow cytometry using FACS Canto II with FACSDiva Software (BD Biosciences, Heidelberg, Germany). Specifically, 300,000 events were collected in the mononuclear cell gate according to the FSC *vs*. SSC. Different subsets of Tcon (CD4^+^FoxP3^−^) and Treg (CD4^+^CD25^+^FoxP3^+^) cells were characterized. Myeloid-derived suppressor cells (MDSC) were characterized as CD11b^+^Gr1^+^. FlowJo software (Tree Star) was used to analyze at least 500,000 events.

### 4.8. *In Vitro* Treatment of Murine Splenocytes

Single cell suspensions of spleens were prepared as described elsewhere [45]. Spleens were homogenized, squeezed through a 100 and 40 μm cell strainer and flushed with 10 mL of PBS to collect as much cells as possible and finally suspended in PBS. The cells were then counted, the density adjusted to 2 × 10^6^/mL and 5 mL of this cell suspension was cultivated in a RPMI-1640 medium with 10% fetal calf serum for 24 h, with or without IFN (5.000 U) + 5-FU (450 μM). After that, the cells were gently scratched and proceeded to FACS staining, as mentioned above.

### 4.9. Analysis of Human Cytokines with LUMINEX

Human serum cytokines (interferon γ (IFNγ), tumor-necrosis factor α (TNF-α), IFNγ-induced protein 10 (IP10), and interleukin 10 (IL10), IL12 and IL17) were determined using a MILLIPLEX^®^ MAP Kit (Millipore GmbH, Schwalbach/TS, Germany) according to the manufacturer’s specifications. Briefly, 25 μL of patients’ sera was incubated overnight at 4 °C with color-coded beads coated with the capture antibodies for the respective cytokines. After triple rinsing, the beads were incubated with the biotinylated secondary detection antibodies for each cytokine/chemokine for one hour at room temperature, followed by the final incubation with streptavidin-phycoerythrin for 30 min at room temperature. Afterwards, triple rinsing measurements were completed using the Luminex^®^ 100/200 System (Millipore, Schwalbach/TS, Germany). According to the standard curves, the concentration of the respective cytokines was calculated and given as pg/mL.

### 4.10. Cytotoxicity Assay

A standard chromium release assay was used to determine the cytotoxic activity, using patients’ PBMC as effector cells and a K562 cell line as a source of target cells. The assay was performed as described previously in [44].

### 4.11. ELIspot

For the ELIspot for granzyme B, the BD™ ELISPOT Human Granzyme B ELISPOT Set (BD Bioscience, San Diego, CA, USA) was used according to the manufacturer’s instructions. Patients’ PBMC were stimulated with 100 mg/mL MUC-1 (self-made as described in [44]) or 100 U/mL CA19.9 (Merck, Schwalbach/TS, Germany), or with the medium as a control. Spots were counted after 24 h of incubation using the KSELISPOT System, release 4.1 (Carl Zeiss Light Microscopy, Göttingen, Germany). Cells stimulated with the medium without the MUC-1 peptide served as controls.

### 4.12. Statistical Analysis

GraphPad Prism Version 5.01 software (Statcon, Witzenhausen, Germany) [46] was used for statistical analyses. Distributions of continuous variables were described by means of SE (standard error), median, 25% and 75% percentiles and were presented as dot plots. D’Agostino and Pearson omnibus normality tests were conducted to estimate the distribution of data. The null hypothesis (mean values were equal at all time points of the treatment) versus the alternative hypothesis (mean values were not equal) was tested by repeated measures of ANOVA, with Bonferroni’s multiple comparison post-hoc test for the normal distributed variants and by Mann-Whitney test for the data that did not pass the normality test. Difference in clinical parameters between the study arms was assessed using the χ-square test. Correlation analyses of nonparametric data were conducted using the Spearman correlation coefficient (*r*). All statistical tests were two-tailed. The significance level was α = 5%.

## 5. Conclusions

An immunomonitoring of 17 patients with pancreatic cancer from the CapRI-2 study, and tumor-bearing mice treated with combination of chemo (radio) therapies with IFN was performed and immunological effects of IFN and chemo-radio-immunotherapy with IFN and 5-FU were analyzed in this study. The data demonstrated an immunomodulatory response to IFN therapy in pancreatic carcinoma patients. Analysis of the Panc02 orthotopic mouse model of pancreatic cancer revealed a collateral accumulation of the MDSC in spleens and tumors of the mice treated with IFN and 5-FU, which was supported by the direct effect of the drugs on MDSC detected *in vitro*. Our human and mouse *in vivo* and *in vitro* studies indicate that the unspecific immunotherapy of pancreatic carcinoma with IFN can induce not only the activation of anti-tumor immunity, but also the immunosuppression. The data demonstrate the importance of immunosuppressive mechanisms induced by combined chemo-immunotherapy and might provide an explanation for the absence of clinical benefit for patients in CapRI study.

## Supplementary Information



## Figures and Tables

**Table 1.** Patients’ characteristics.

Arm/Treatment	Patient	Gender	Age	Resection Type	Tumor Grade	TNM	Relapse/Location	DFS *, Month	Status	OS **, Month
A/	A1	M	52	R1	G2	pT3, pN1 (7/32), pMx	Liver	15	Dead	16
IFN	A2	F	47	R1	G2	pT3, pN1 (2/24), pMx	0	20	Alive	20
5FU	A3	M	58	R1	G2	pT3, pN1(8/49), pMx	Liver	4	Dead	12
EBR	A4	M	50	R1	G2	pT2, pN1(1/6), M0	Peritonealcarcinosis	18	Dead	23
Cis	A5	M	67	R1	G2	pT3, pN1(1/19), M0	0	51	Alive	51

B/	B1	M	64	R1	G2	pT3, pN1 (6/22), pMx	Liver	3	Alive	63
IFN	B2	F	69	R0	G2	pT3, pN1 (1/37), pMx	0	63	Alive	63
5FU	B3	F	60	R1	G2	pT3, pN1 (3/28), pMx	Liver	15	Dead	21
EBR	B4	M	65	R0	n.d. ***	pT3 pN1 (1/17), cM0	0	53	Alive	53
	B5	M	54	R1	G2	pT3 pN1 (18/58), cM0	Peritonealcarcinosis	17	Dead	48
	B6	M	62	R1	G3	pT3, pN1(6(35), pMx	Peritonealcarcinosis	4	Dead	7

C/	C1	F	67	R1	G2	pT3, pN1(3/24), M0	Liver	27	Dead	48
IFN	C2	M	63	R1	G2	pT3, pN1 (10/29), Mx	0	47	Alive	47
5FU	C3	M	59	R1	G2	pT3, pN1 (5/29), pMx	Liver	12	Dead	20
	C4	F	69	R1	G2	pT3, pN1(5/21), pMx	Liver	3	Dead	7
	C5	M	62	R1	G2	pT3 pN1 (13/27), M0	Liver	19	Dead	38
	C6	F	65	R1	G3	pT3, pN0 (0/43), M0	Peritonealcarcinosis	4	Dead	7

* DFS—disease free survival;

** OS—overall survival;

*** n.d.—no data.

**Table 2.** Spearman correlation of values CD4^+^ cells and MDSC (myeloid-derived suppressor cells) against tumor growth.

		CD4^+^ Cells		MDSC	
			
		*r*	*p*	*r*	*p*
Tumor growth	co	−0.94	0.017	0.83	0.058
	IFN	−0.7	0.233	0.54	0.297
