# Supplementary material for: Influence of Interferon-Alpha Combined with Chemo (Radio) Therapy on Immunological Parameters in Pancreatic Adenocarcinoma
Source: Int J Mol Sci. 2014 Mar 7;15(3):4104–25. doi: 10.3390/ijms15034104 (PMC3975387; doi:10.3390/ijms15034104)
Supplement: Supplementary file 1 [file ijms-15-04104-s001.pdf]

Supplementary Information

Figure S1. Representative FACS picture of monocyte and DC analysis of one patient before and during IFN therapy. The percentage from total leukocytes is notified.

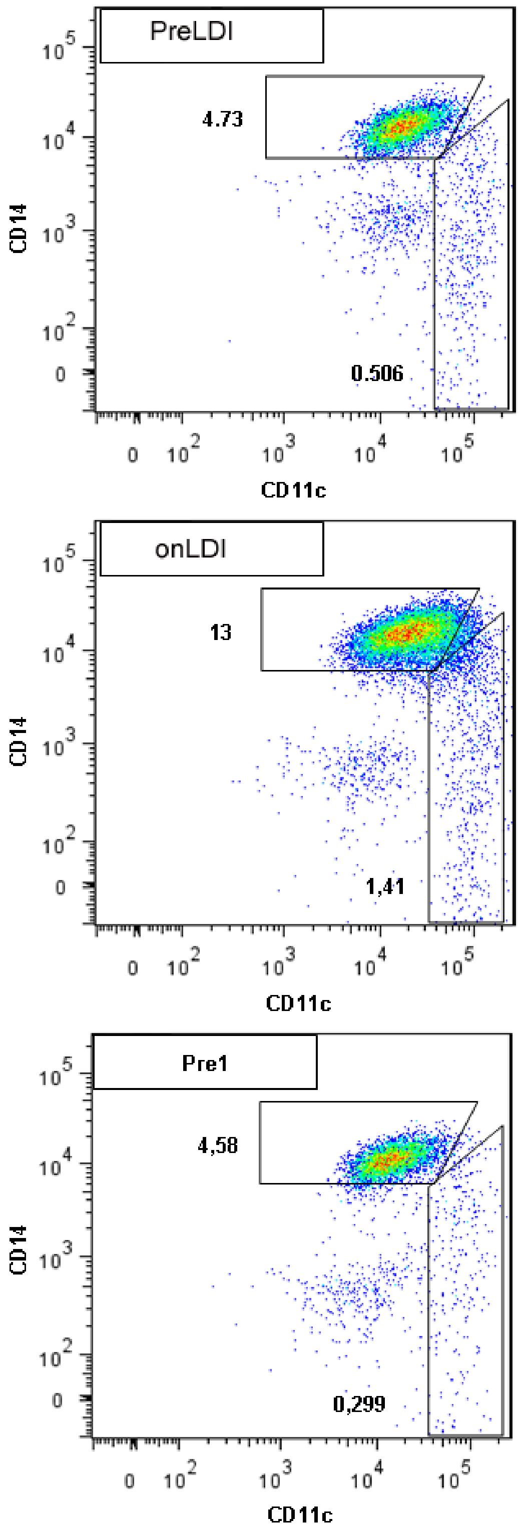

Figure S2. Representative pictures of FACS analysis of CD86 (**left**) and HLA-DR (**right**) expression of one patient before and during IFN therapy.

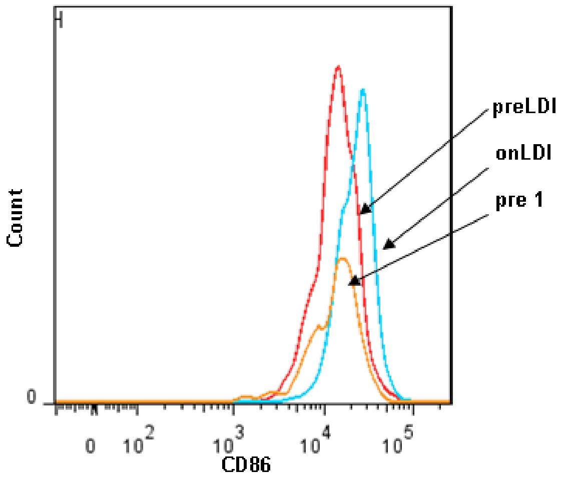

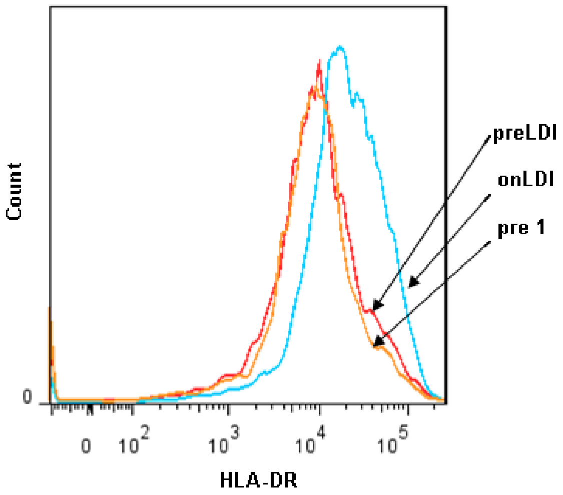

Figure S3. Expression of CTLA4 on the surface of $CD4^+$ cells during the course of IFN therapy. Immunological parameters of 17 patients were analyzed.

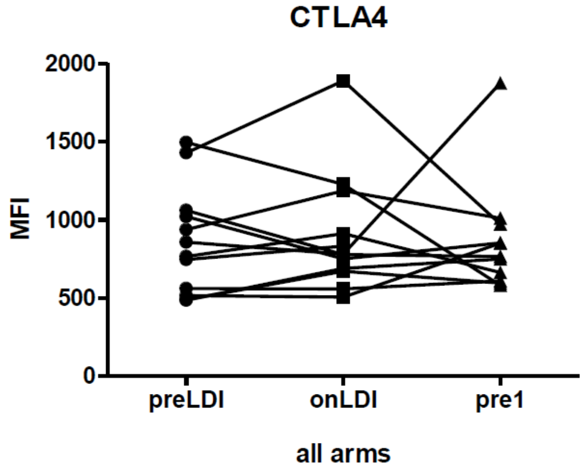

Figure S4. Representative pictures of FACS analysis of NKG2D on all NK cells (**upper panel**), on CD8− NK cells (**middle panel**) and on CD8+ NK cells (**low panel**) of one patient before and during IFN therapy.

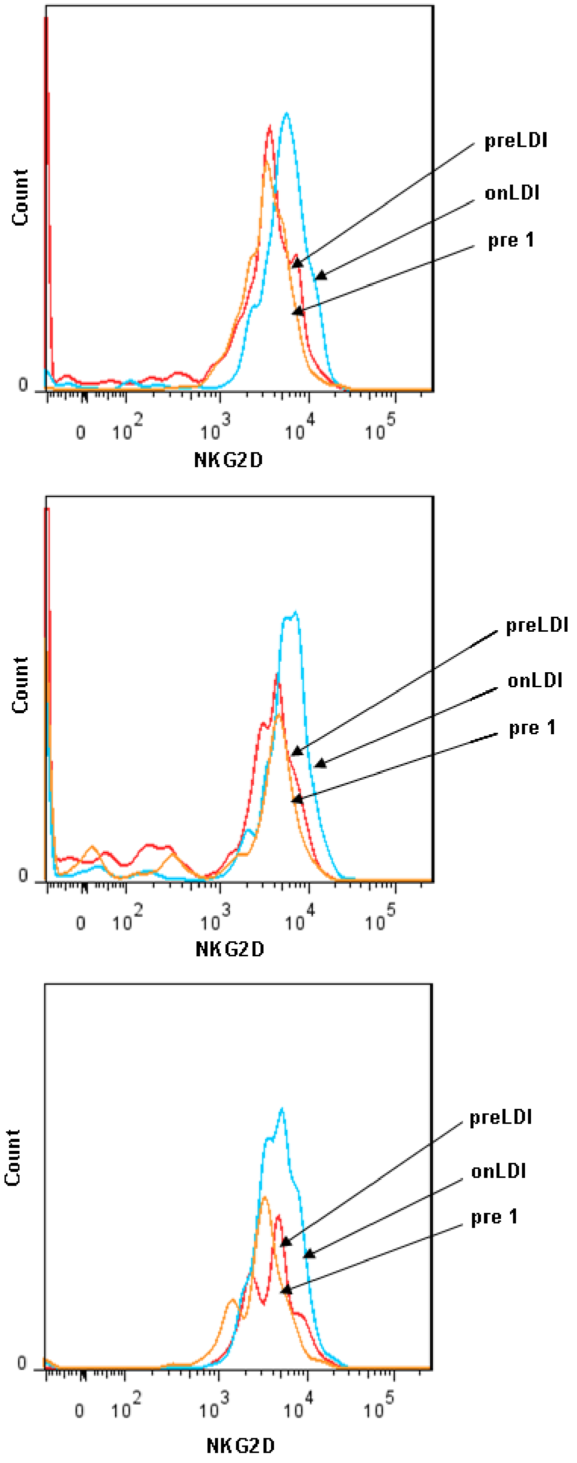

Figure S5. Leukocytes in all arms during the course of chemo-radio-immunotherapy. (**a**) Leukocyte count (clinical chemistry), no data for onLDI and end time-points; (**b**) FACS analysis of monocytes; for (**a**) and (**b**): Arm A *n* = 5, Arm B and C *n* = 6; (**c**) Relative amount of lymphocytes in all arms during the chemo-immunotherapy/ chemo-radio-immunotherapy, *n* = 17, *, $p < 0.05$.

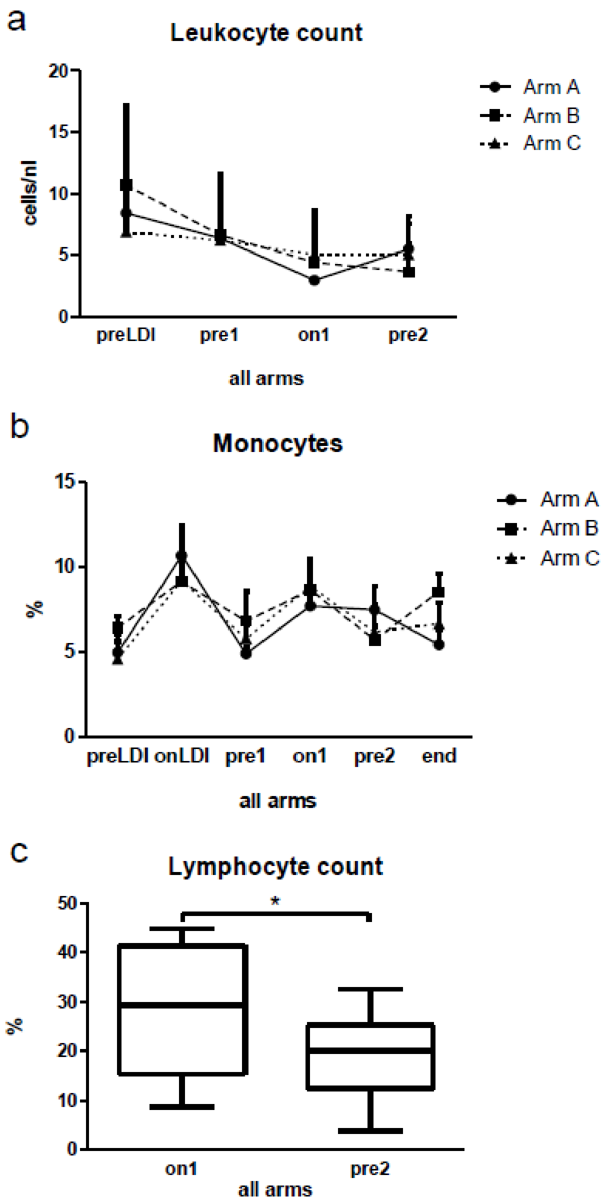

Figure S6. NK-cells: Expression of NKG2D on the surface of all $CD45^+CD56^+$ cells (**a**); $CD45^+CD56^+CD8^-$ cells (**b**); and $CD45^+CD56^+CD8^+$ cells (**c**) in all arms during the course of chemo-radio-immunotherapy. Arm A *n* = 5, Arm B and C *n* = 6.

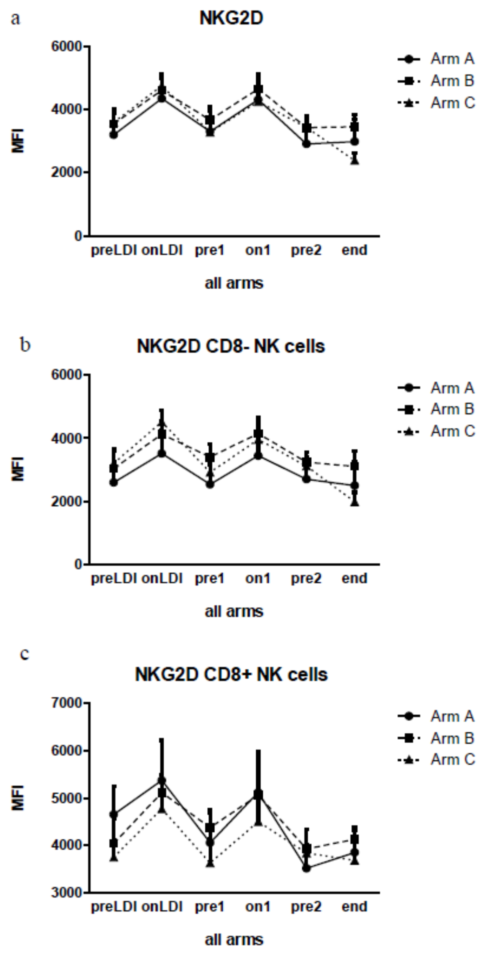

Figure S7. Tumor growth in Panc02 tumor-bearing mice treated with a combination of IFN and 5-FU. Six animals per group were analyzed, *, $p < 0.05$.

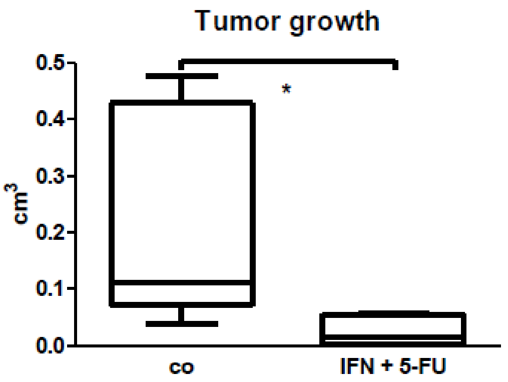

Table S1. Difference in patients' characteristics between study arms.

Arm	Number of patients	Gender			Resection			Tumor grade			TNM			TNM			Relapse			2-y survivor		5-y survivor	
		M	F	*p*	R0	R1	*p*	G2	G3	*p*	T2	T3	*p*	N0	N1	*p*	yes	no	*p*	*n* (%)	*p*	*n* (%)	*p*
A	5	4	1	0.89	0	5	0.39	5	0	0.89	1	5	0.69	0	1	0.47	3	2	0.94	1 (20)	0.49	0	0.22
B	6	4	2		2	4		4	1		0	6		0	6		4	2		5 (83)		2 (33)	
C	6	3	3		0	6		5	1		0	6		1	5		5	1		3 (50)		0	

© 2014 by the authors; licensee MDPI, Basel, Switzerland. This article is an open access article distributed under the terms and conditions of the Creative Commons Attribution license (http://creativecommons.org/licenses/by/3.0/).
